# Single-Walled Carbon Nanotube Dominated Micron-Wide Stripe Patterned-Based Ferroelectric Field-Effect Transistors with HfO_2_ Defect Control Layer

**DOI:** 10.1186/s11671-018-2534-1

**Published:** 2018-04-27

**Authors:** Qiuhong Tan, Qianjin Wang, Yingkai Liu, Hailong Yan, Wude Cai, Zhikun Yang

**Affiliations:** 1 School of Energy and Environment Science, Yunnan Normal University, Yunnan, Kunming, 650500 China; 2 Yunnan Provincial Key Laboratory for Photoelectric Information Technology, Yunnan Normal University, Yunnan, Kunming, 650500 China; 3 College of Physics and Electronic Information, Yunnan Normal University, Yunnan, Kunming, 650500 China; 4 College of Physics and Electronic Engineering, Xinyang Normal University, Xinyang, 464000 China

**Keywords:** Ferroelectric field-effect transistors (FeFETs), Single-walled carbon nanotube (SWCNT), Nonvolatile memory, Ferroelectric film

## Abstract

Ferroelectric field-effect transistors (FeFETs) with single-walled carbon nanotube (SWCNT) dominated micron-wide stripe patterned as channel, (Bi,Nd)_4_Ti_3_O_12_ films as insulator, and HfO_2_ films as defect control layer were developed and fabricated. The prepared SWCNT-FeFETs possess excellent properties such as large channel conductance, high on/off current ratio, high channel carrier mobility, great fatigue endurance performance, and data retention. Despite its thin capacitance equivalent thickness, the gate insulator with HfO_2_ defect control layer shows a low leakage current density of 3.1 × 10^−9^ A/cm^2^ at a gate voltage of − 3 V.

## Background

Ferroelectric field-effect transistor (FeFET) is a promising candidate for nonvolatile memory devices and integrated circuits because of its high speed, single device structure, low power consumption and nondestructive read-out operation [1–3]. (Bi,Nd)_4_Ti_3_O_12_ (BNT) is a Pb-free ferroelectric thin film with stable chemical properties and fatigue endurance performance. Thus, the FeFET using BNT as the gate dielectric would have smaller threshold voltage, large channel conductance, and so on. Carbon nanotubes (CNTs) have been widely applied in FeFET for its high conductivity and large carrier mobility [4–7]. It is well known that there are no dangling bonds on the surface of ideal CNTs, which leads to small interface reaction between ferroelectric film and CNTs [8, 9]. However, it is very difficult to achieve single CNT growth between source and drain electrodes in experiment. Besides, the on/off current ratio of CNT nanowire network FeFET is generally low because of the admixture of metallic nanotubes in CNT network [7, 10]. Song et al. proposed to use multiwalled CNT micron-wide stripe patterned as channel material of FeFET to solve the abovementioned problems, but the fatigue endurance performance and retention of physical characteristics of CNT FeFET is not clear [9]. Compared to multiwalled CNT (MWCNT), the single-walled CNT (SWCNT) is a seamlessly wrapped single graphene sheet formed into a cylindrical tube [11]. Moreover, there are some defects (such as ion impurities, oxygen vacancies, and dislocations) which are difficult to control during the fabrication of ferroelectric thin film [12–14]. The diffusion of these defects can affect the on/off current ratio, fatigue endurance performance, and data retention [15, 16]. Therefore, we implant HfO_2_ layer in SWCNT-FeFET, which is used to block diffusion of point defects and can be used as a buffer layer to relieve the misfit between BNT and Si and therefore to reduce the dislocation density in the BNT film. It can control the defects in SWCNT-FeFET, and then significantly improve the on/off current ratio, fatigue characteristics, and data retention.

**Fig. 1:**
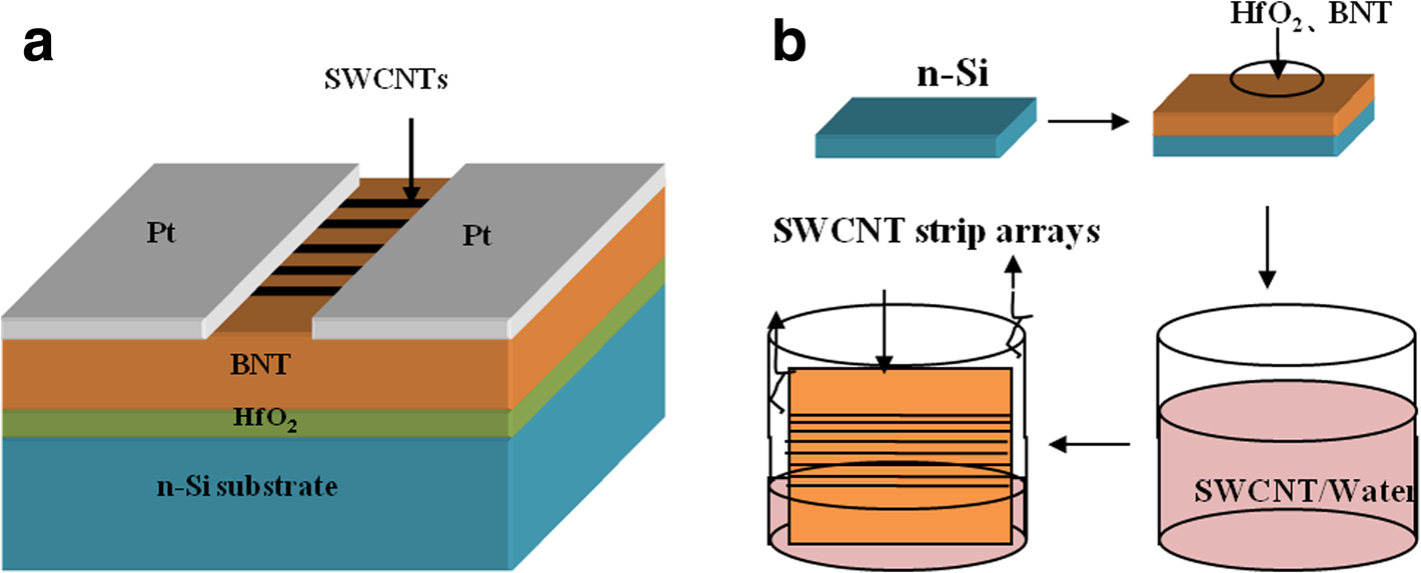
**a** The structure diagram of the stripe patterned SWCNT-FeFET. **b** Flow chart of the stripe patterned SWCNT/BNT/HfO_2_-FeFET fabrication

In this study, we fabricated regular and aligned micron-wide stripe patterned network SWCNTs as channel layer, BNT films as insulator, and HfO_2_ films as defect control layer to fabricate bottom-gate type FeFET and expected to obtain good on/off current ratio, fatigue characteristics, and data retention. The structure of SWCNT-FeFET and its preparation procedure are shown in Fig. 1a, b. Besides, we have also fabricated MWCNT-FeFET for comparison.

## Methods

In the FeFET devices, the SWCNT micron-wide stripe patterned is used as channel, the BNT thin film is used as gate dielectric, HfO_2_ films are used as defect control layer, and the heavily doped n-type Si is used as substrate and back gate electrode of FeFET simultaneously. The resistivity of n-type Si is 0.0015 Ω cm. The HfO_2_ was deposited on the Si substrate by pulsed laser deposition (PLD) using a KrF excimer laser with a wavelength of 248 nm, and its thickness is about 20 nm. The BNT film was deposited on the Si substrate by PLD as described in the early work [17], and its thickness is about 300 nm. The pristine arc-discharged SWCNT was purchased from Chengdu Institute of Organic Chemistry (Chinese Academy of Science); the length and diameter are 10–30 μm and 0.8–1.1 nm, respectively. Its purity is 85% which signifies that SWCNT is dominated. The SWCNTs were fabricated by using evaporation-induced self-assembly. The concentration of SWCNT/water dispersion was 100 mg/L, the evaporation rate was varied in a range of 9–21 μL/min, and the temperature was 80 °C. By controlling the solvent evaporation temperature, well-defined stripe pattern was formed at the solid-liquid-vapor interface on the BNT/HfO_2_/Si substrate. Next, Pt source/drain electrodes were deposited on the SWCNTs/BNT by ion-beam sputtering using a mask plate. The total area of the metal mask plate is 1 cm^2^, and the areas of source and drain are both 4.5 mm^2^. The channel length (*L*) and width (*W*) of FeFET are 200 and 1500 μm, respectively. The fabricated SWCNT-FeFET followed by a post annealing at 500 °C for 2 h to improve the contact between source/drain electrodes and SWCNTs. As reported, the CNT network contains both metallic and semiconducting nanotubes. The CNT network was processed by applying a large gate voltage. The metallic SWCNT nanotubes were nearly ablated and semiconducting SWCNT nanotubes were remained by load current [18]. In order to compare, SWCNT/SiO_2_-FET were fabricated by the same method and conditions; MWCNTs/BNT-FET was also fabricated by the method as described in the early work [9]. FeFET characteristics were measured using a Keithley 4200 parameter analyzer. The hysteresis loops and polarizations of FeFET were measured using a RT Precision Workstation ferroelectric analyzer.

## Results and Discussion

**Fig. 2:**
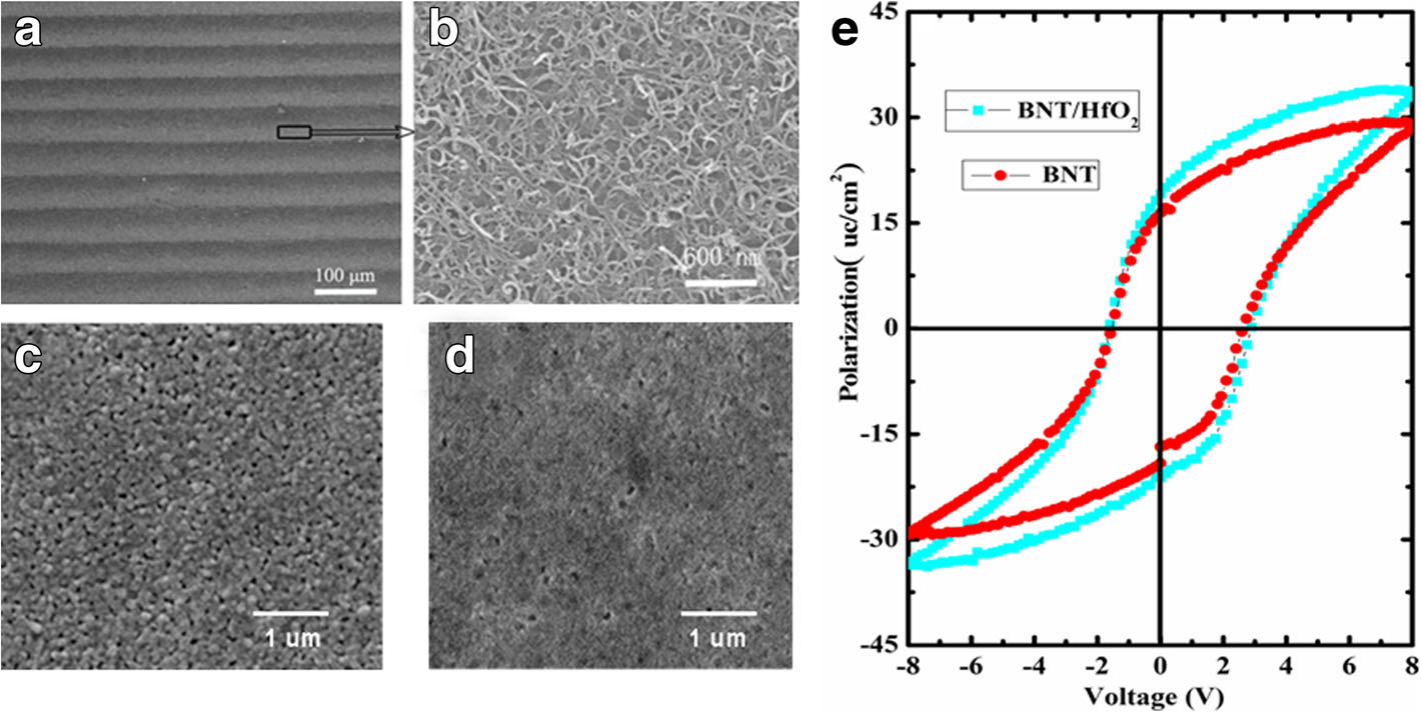
**a** SEM micrograph of SWCNT stripe patterned in the SWCNT/BNT/HfO_2_-FeFET. **b** The grid structure of SWCNTs. **c** SEM image of the surface for BNT/HfO_2_ film. **d** SEM image of the surface for BNT film. **e** Hysteresis loops of BNT and BNT/HfO_2_ films

Figure 2 shows typical SEM images of the SWCNTs stripe patterned. The regular and aligned SWCNTs micron-wide stripe patterned are displayed in Fig. 2a. The protuberant and light stripes are SWCNT stripes, in which SWCNTs are densely packed as exhibited in the magnified image of a stripe in Fig. 2b. The sunken and gray stripes correspond to the bared BNT/HfO_2_/Si substrate in the spaces between SWCNT micron-wide stripes. The concentration of SWCNT precursor solution is increased with evaporating, and the width of graded stripes slightly increases with declining of the SWCNT/water liquid level. The BNT/HfO_2_ films and BNT films on the Si substrate are shown in Fig. 2c, d. It can be seen that the surface of the BNT/HfO_2_ film is composed of many crystalline grains and pores, which indicated larger roughness than that of the BNT films. Figure 2e shows the *P*-*V* hysteresis loops of BNT and BNT/HfO_2_ films, respectively. The polarizations of hysteresis loops of the BNT/HfO_2_ films are larger than that of BNT films in the same voltage. Even though HfO_2_ layer shared part of the voltage of BNT/HfO_2_ films, the BNT film still shows better polarization value than that of BNT grown on Si substrate. It is because the BNT films grown on the HfO_2_ layer have lower diffusion defect concentration than that of BNT films grown on the Si substrate directly.

**Fig. 3:**
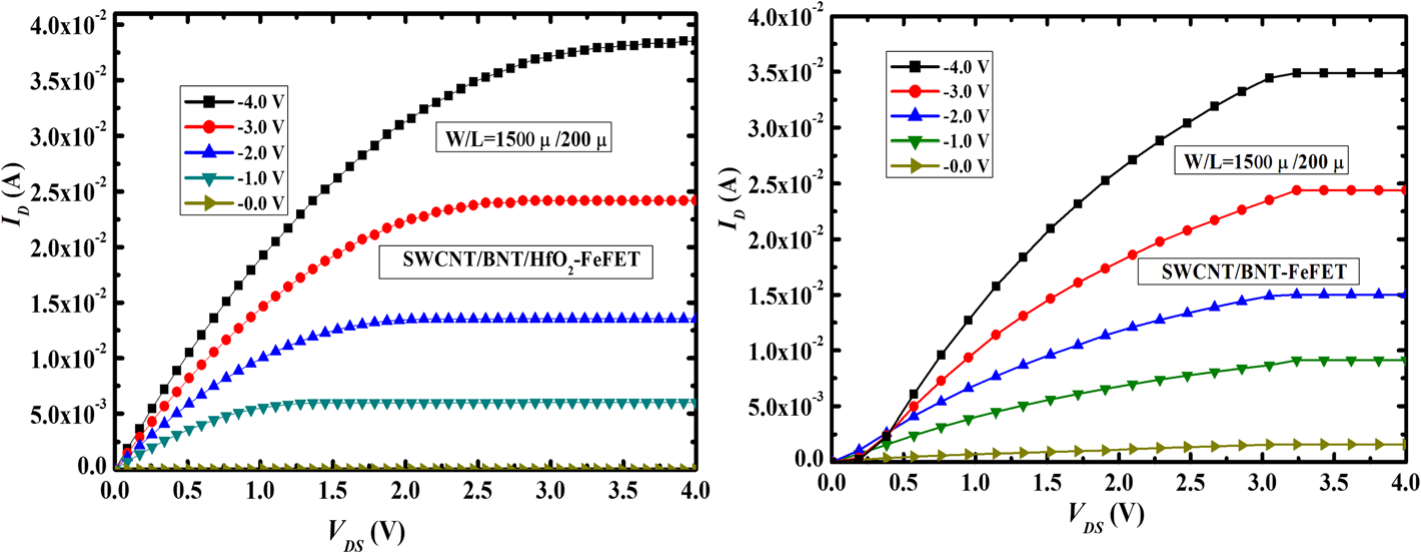
Output characteristics curves of SWCNT/BNT/HfO_2_-FeFET(letf) and SWCNT/BNT-FeFET(right)

Figure 3 shows the output characteristics (*I*_DS_*-V*_DS_) curves of SWCNT/BNT/HfO_2_-FeFET and SWCNT/BNT-FeFET. SWCNT/BNT/HfO_2_-FeFET and SWCNT/BNT-FeFET show the typical *p*-channel transistor characteristics and saturated source-drain currents at low source-drain voltage. Their channel length (*L*) is 200 μm. Because of SWCNT micron-wide stripe patterned, the “on” current and channel conductance of SWCNT/BNT/HfO_2_-FeFET and SWCNT/BNT-FeFET both reach to 3.8×10^−2^ A, 3.6×10^−2^ A and 9.5×10^−3^ S, 9×10^−3^ S at *V*_GS_ = − 4 V and *V*_DS_ = 4 V. However, SWCNT/BNT/HfO_2_-FeFET shows lower off-state currents than that of SWCNT/BNT-FeFET, and SWCNT/BNT-FeFET shows serious leakage current phenomenon at *V*_GS_ = 0 V_._ This is because the HfO_2_ layer effectively blocks the diffusion of defects.

**Fig. 4:**
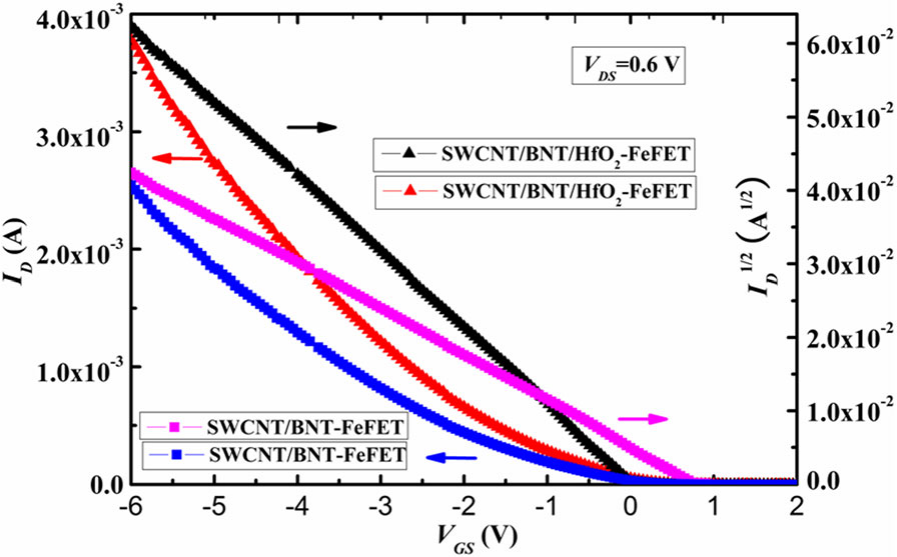
The linear transfer characteristics curve and fitted *I*_DS_^1/2^-*V*_G_ curve at *V*_DS_ = 0.6 V for the stripe patterned of SWCNT/BNT/HfO_2_-FeFET and SWCNT/BNT-FeFET

**Fig. 5:**
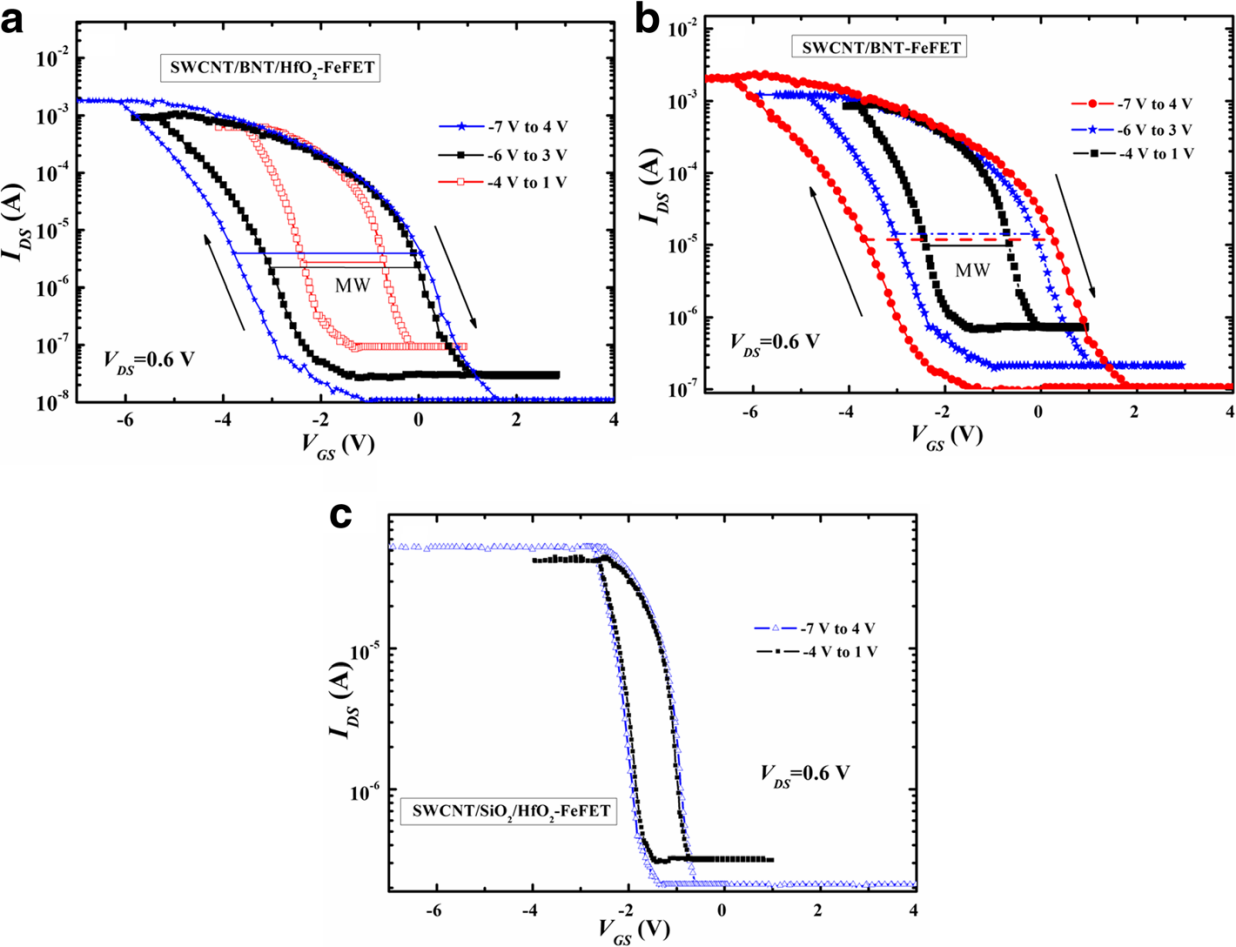
Logarithmic transfer curves of stripe patterned **a** SWCNT/BNT/HfO_2_-FeFET, **b** SWCNT/BNT-FeFET, and **c** SWCNT/SiO_2_/HfO_2_-FET at *V*_*DS*_ = 0.6 V_._ The arrows indicate an anticlockwise hysteresis loop, and the solid lines exhibit the width of memory window

The transfer characteristics (*I*_D_-*V*_G_) of the SWCNT/BNT/HfO_2_-FeFET and SWCNT/BNT-FeFET with *L* = 200 μm and *V*_DS_ = 0.6 V are exhibited in Fig. 4. The threshold voltage (*V*_th_) of SWCNT/BNT/HfO_2_-FeFET and SWCNT/BNT-FeFET are *V*_th_ = 0.2 V and *V*_th_ = 0.8 V by a linear fit of the (*I*_*D*_)^1/2^ vs *V*_GS_ curve of the transistor operating in the saturation region. The channel mobility (*μ*_sat_) is calculated based on the (*I*_DS_)^1/2^ vs *V*_GS_ curve as well as the saturation region expression for a field effect transistor [19],

$$ {I}_{\mathrm{DS}}=\left(\frac{\varepsilon_0{\varepsilon}_r{\mu}_{\mathrm{sat}}W}{t_{\mathrm{ins}} 2L}\right){\left({V}_{\mathrm{GS}}\hbox{-} {V}_{\mathrm{th}}\right)}^2 for\kern0.5em {V_{\mathrm{DS}}}^{{}^3}{V}_{\mathrm{GS}}\hbox{-} {V}_{\mathrm{th}}, $$

where *ε*_*r*_ is the relative permittivity and *t*_ins_ is the BNT thickness. A relative dielectric constant (*ε*_*r*_) of BNT film is 350, which is measured at 1 MHz by HP4156 parameter analyzer. The *μ*_sat_ of SWCNT/BNT/HfO_2_-FeFET and SWCNT/BNT-FeFET are 395 and 300 cm^2^/V s. Figure 5 shows the *I*_DS_–*V*_GS_ logarithmic transfer curves of the fabricated SWCNT/BNT/HfO_2_-FeFET, SWCNT/BNT-FeFET, and SWCNT/SiO_2_/HfO_2_-FET in a double sweep mode. The gate voltage sweep was performed at a *V*_DS_ of 0.6 V and at the *V*_GS_ ranges from *−* 7 to 4 V, *−* 6 to 3 V, and *−* 4 to 1 V. The *I*_DS_ on/off ratio of SWCNT/BNT/HfO_2_-FeFET, SWCNT/BNT-FeFET, and SWCNT/SiO_2_/HfO_2_-FET are 2 × 10^5^, 2 × 10^4^, and 2.3 × 10^2^ at the *V*_GS_ range from *−* 7 to 4 V. The *I*_DS_ on/off ratios of SWCNT/BNT/HfO_2_-FeFET are higher than that of MWCNT/BNT-FeFET [9] and SWCNT/BNT-FeFET. It is because the HfO_2_ defect control layer was implanted in SWCNT-FeFET, which effectively blocks the diffusion of defects. For *I*_DS_-*V*_GS_ characteristics, we obtained a clockwise hysteresis loop owing to the ferroelectric polarization reversal of the BNT film, the obtained memory window (MW) width of SWCNT/BNT/HfO_2_-FeFET and SWCNT/BNT-FeFET are about 4.2 and 4.1 V, which is larger than that of CNT/PZT-FeFET (1.1 V) with the CNT network as channel layer [20]. The larger MW indicates good dielectric coupling in this FeFET system. From Fig. 4c, we can see the obtained window width of SWCNT/SiO_2_/HfO_2_-FET is about 1 V, which is mainly caused by defect densities of SWCNT [21]. These results suggest that the memory window hysteresis (4.2 V) of ferroelectric FeFET is caused by both BNT polarization and densities of SWCNT defects.

**Fig. 6:**
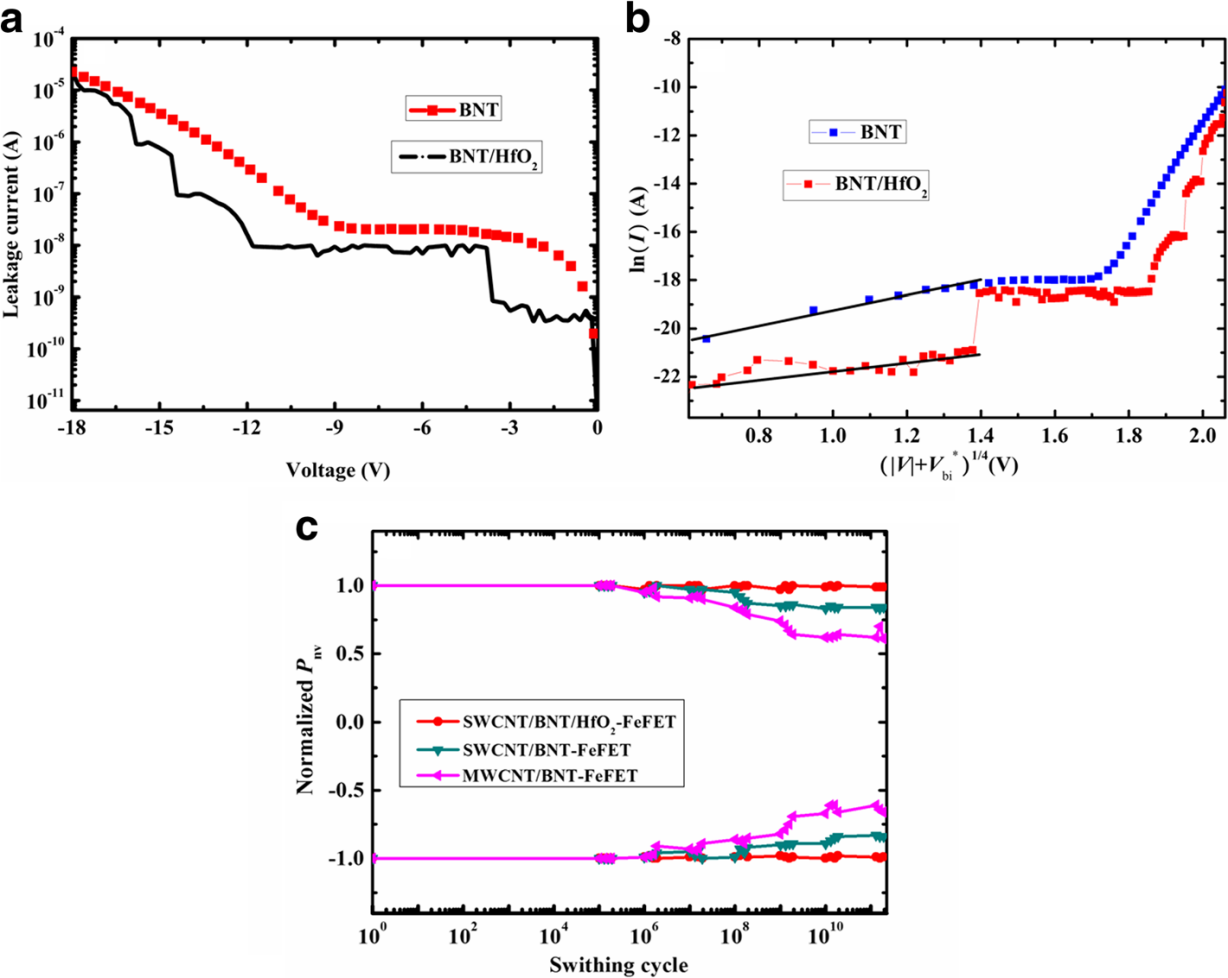
**a** Leakage current-voltage characteristics of the BNT/HfO_2_ and BNT film. **b** The fitting curve of the leakage current-voltage characteristics of the BNT/HfO_2_ and BNT film. **c** Fatigue endurance performance of the SWCNT/BNT/HfO_2_-FeFET, SWCNT/BNT-FeFET, and MWCNT/BNT-FeFET

Figure 6a shows the leakage current-voltage characteristics of the BNT/HfO_2_ and BNT film. As can be seen, the leakage currents are 1.2 × 10^−9^ A and 1.5 × 10^−8^ A for BNT/HfO_2_ and BNT film, respectively, when the voltage reaches up to − 3 V. The leakage current-voltage characteristics of the BNT/HfO_2_ and BNT film were studied for comparison by fitting the *I*-*V* data. The leakage current characteristics of a Schottky contact were represented by Ln(*J*) = *b*(*V + V*_bi_^*^)^1/4^ [9, 22, 23], and the corresponding plots for BNT/HfO_2_ and BNT films in the voltage range of 0 to 3.8 V are shown in Fig. 6b. The built-in voltages *V*_bi_^*^ and slope *b* in the formula can be obtained by fitting the experiment *I-V* data. The calculated space-charge densities *N*_eff_, which consisted of deep trapping centers and oxygen vacancies [22], are about 2.132 × 10^17^ cm^−3^ and 1.438 × 10^19^ cm^−3^ for BNT/HfO_2_ and BNT film, respectively. It is indicated that the BNT films deposited on Si substrate are n-type semiconductors according to the formula of interface barrier heights [24]. This is consistent with effect of the HfO_2_ on reducing the off-current of *I*_D_-*V*_G_ curve in Fig. 4a, b, because n-type BNT generate electron increases the off-current in negative voltage. BNT film conduction shows bulk-controlled mechanism, which further implies that the n-type BNT is mainly induced by the conductive defects or impurities [9, 22].

Figure 6c shows the fatigue endurance performance for the SWCNT/BNT/HfO_2_-FeFET, SWCNT/BNT-FeFET, and MWCNT/BNT-FeFET with a 100-KHz bipolar pulse at the *V*_GS_ range from *−* 7 to 4 V. The fatigue endurance performance of FeFET is exhibited in the loss of switchable polarization with repeated switching cycles. The value of non-volatile polarization (*P*_nv_) is obtained by the equation *P*_nv_ = *P*_r_^*^ − *P*_r_^^^ and then, normalized with *P*_nv_/*P*_r0_^*^ [25], where *P*_r_^*^ is twice remnant polarization of FeFET, *P*_r_^^^ is the loss of polarization after the next pulse, and *P*_r0_^*^ is the twice initial remnant polarization of FeFET. The partial loss of the normalized *P*_nv_ after 10^11^ read/write switching cycles is observed for the FeFET, which are approximately 3, 10, and 25% for SWCNT/BNT/HfO_2_-FeFET, SWCNT/BNT-FeFET, and MWCNT/BNT-FeFET, respectively. When BNT directly grows on the bottom electrode Si, the fatigue performance of SWCNT/BNT-FeFET is very bad because of the diffusion between BNT and Si substrate through grain boundary [12–14]. These results suggest that the HfO_2_ layer effectively blocks the diffusion of Si substrate and reduces the ion impurities, which results in excellent fatigue endurance performance.

**Fig. 7:**
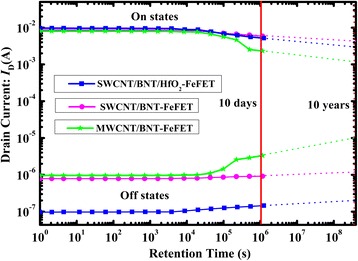
Retention characteristics of the SWCNT/BNT/HfO_2_-FeFET, SWCNT/BNT-FeFET and MWCNT/BNT-FeFET at room temperature

To assess the device reliability of FeFET toward NVRAM application, we have examined data retention. Figure 7 shows the source-drain current retention curves for the SWCNT/BNT/HfO_2_-FeFET, SWCNT/BNT-FeFET, and MWCNT/BNT-FeFET at room temperature. The voltage pulse of *V*_GS_ = − 4 V and *V*_GS_ = 1 V at *V*_DS_ = 1 V is applied to the gate and source-drain electrode, switching the voltage of FeFET to off or on state, respectively. The measured on/off-state current ratios are nearly 3 × 10^4^, 7 × 10^3^, and 6 × 10^2^ for SWCNT/BNT/HfO_2_-FeFET, SWCNT/BNT-FeFET, and MWCNT/BNT-FeFET after 10^6^ s, respectively. There is no significant loss in on/off-state current ratio (3.2%) after a retention time of 1 × 10^6^ s for SWCNT/BNT/HfO_2_-FeFET. By extrapolating the curves to 10^8^ s for SWCNT/BNT/HfO_2_-FeFET, SWCNT/BNT-FeFET, and MWCNT/BNT-FeFET, the on/off-state current ratios are nearly 1.9 × 10^4^, 3 × 10^3^, and 2 × 10^2^, respectively. The on/off-state ratio of SWCNT/BNT/HfO_2_-FeFET is still high enough for the function of memories, demonstrating a desirable retention property of the present memory device. Retention is influenced by the gate leakage current [26, 27]. The long retention time indicates HfO_2_ defect control layer can effectively reduce the off-state current and gate leakage current, which stabilizes the on/off current ratio. In addition, we also made a comparison between ferroelectric-based FETs and different CNT in Table 1, suggesting that the fabricated SWCNT/BNT/HfO_2_-FeFET in this study can provide high on/off current ratio, great fatigue endurance performance, and data retention.

**Table 1:** Comparison of ferroelectric-based FETs with different CNT

FeFET structure	Arrays SWCNT/BNT/HfO_2_ [this work]	Arrays SWCNT/BNT [this work]	Single SWCNT/BTO Ref. [6]	Arrays MWCNT/BNT Ref. [8]	Networked SWCNT/PVD Ref. [3]
Fabrication method	Evaporation/sol-gel/sol-gel	Evaporation/sol-gel	PECVD/PLD	Evaporation/sol-gel	Dispersion/spin-coated
Channel length (μm)	200	200	0.6	200	100
Operating voltage (V)	2	4	1	4	30
“On” current	3.8 × 10^−2^	3.6 × 10^−2^	7 × 10^−7^	1.5 × 10^−2^	10^−6^
*V*_th_ (V)	0.2	0.8	2.5	1.6	15
*μ*_sat_(cm^2^/V-s)	395	300	260	94.47	–
MW (cm)	4.2 (logarithmic)	4.1 (logarithmic)	4 (logarithmic)	4.38 (linear)	–
On/off current ratio	2 × 10^5^	2 × 10^4^	10^3^	10^3^	10^4^
Fatigue characteristics (10^11^)	Less 3%	10%	–	25% [this work]	–
Data retention (10^6^s)	3 × 10^4^	7 × 10^3^	7 × 10^2^	6 × 10^2^ [this work]	–

**Fig. 8:**
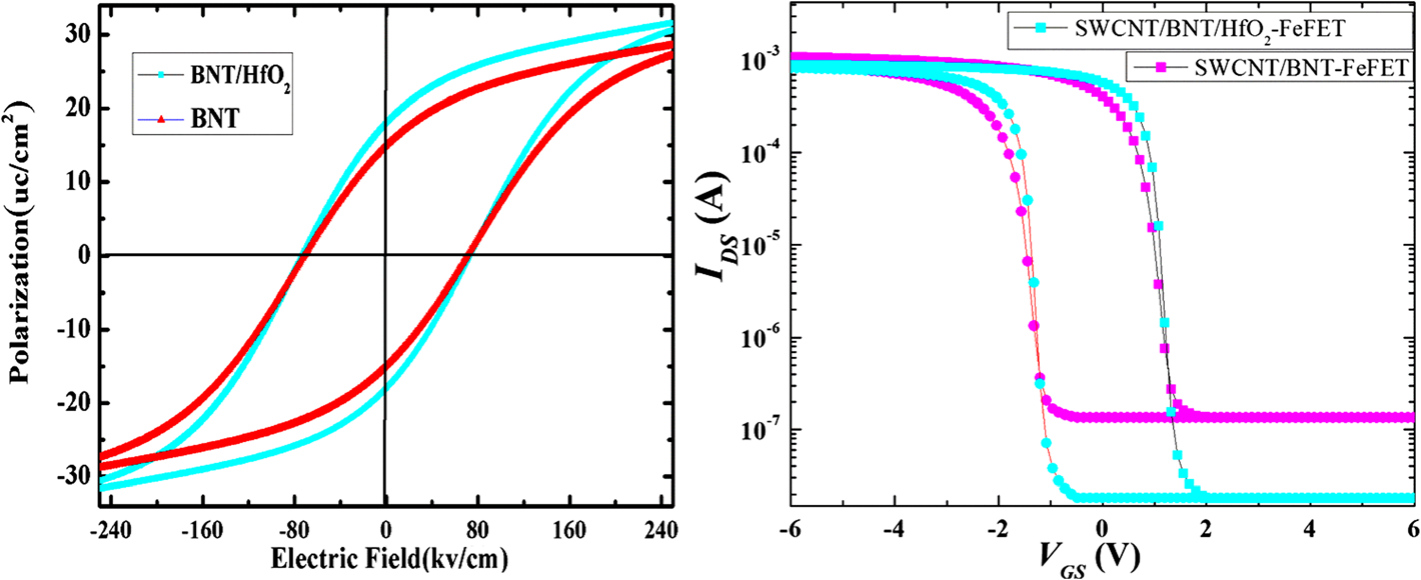
Simulation of **a** *P*-*E* hysteresis loops of BNT and BNT/HfO_2_ films and **b** *I*_DS_-*V*_GS_ curve for the SWCNT/BNT/HfO_2_-FeFET and SWCNT/BNT-FeFET

In order to further understand how the defects influence the physical characteristics of the device, the *P*-*E* hysteresis loops and *I*_DS_-*V*_GS_ curve for the SWCNT/BNT/HfO_2_-FeFET and SWCNT/BNT-FeFET were simulated by considering asymmetric charge caused by defects using our previous models [12, 28]. An asymmetric charge caused by defects is considered to simulate the *P*-*E* hysteresis loops and *I*_DS_-*V*_GS_ curve of BNT, and a symmetrical charge is considered to simulate that of BNT/HfO_2_. The simulation results are shown in Fig. 8a, b, which are similar with the experimental results of Figs. 2e and 5a, b, respectively. The simulation results indicate HfO_2_ layer effectively reduces the asymmetric charges of ferroelectric films caused by defects, which further increases the off-state current. Therefore, it can be demonstrated that the defects of ferroelectric thin film were effectively controlled by HfO_2_ defect control layer.

## Conclusions

In summary, the effect of HfO_2_ materials as defect control layer on the on/off current ratio, fatigue endurance performance, and data retention of the SWCNT/BNT-FeFETs has been investigated, in which the defects of ferroelectric thin film are controlled by HfO_2_ as the defect control layer. Due to the thin defect control layer of HfO_2_, the fabricated SWCNT/BNT/HfO_2_-FeFET shows a low leakage current of 1.2 × 10^−9^ A when the voltage reaches to − 3 V, a large on/off current ratio of 2 × 10^5^, a *V*_th_ of 0.2 V, and a *μ* of 395 cm^2^/V s. Moreover, the SWCNT/BNT/HfO_2_-FeFET showed excellent fatigue endurance performance and good data retention that are attributed to the thin HfO_2_ defect control layer. The hysteresis loops *P*-*E* and *I*_DS_-*V*_GS_ curve for the SWCNT/BNT/HfO_2_-FeFET and SWCNT/BNT-FeFET were simulated to understand how the defects influence the physical characteristics of the device. The simulation results further demonstrated the asymmetric charge can be reduced in SWCNT/BNT/HfO_2_-FeFET by HfO_2_ to control the defects.

## Abbreviations

BNT: (Bi,Nd)_4_Ti_3_O_12_
FeFETs: Ferroelectric field-effect transistors
MWCNT: Multiwalled CNT
PLD: Pulsed laser deposition
SWCNT: Single-walled carbon nanotube
